# Assessment of anatomical outcomes in the upper urinary tract following flexible ureteroscopy with flexible and navigable suction ureteral access sheath: 1‐year results from a multicentre study

**DOI:** 10.1002/bco2.70213

**Published:** 2026-05-04

**Authors:** Steffi Kar Kei Yuen, Chi‐Fai Ng, Khi Yung Fong, Bhaskar Kumar Somani, Boyke Soebhali, Mohamed Elshazly, Vigen Malkhasyan, Karl Tan, Luis Rico, Mehmet Ilker Gokce, Han Jie Lee, Nariman Gadzhiev, Tzevat Tefik, Jianwei Cao, Albert El Hajj, Chi‐Ho Leung, Jeremy Yuen Chun Teoh, Oliver Traxer, Ben Hall Chew, Daniele Castellani, Vineet Gauhar

**Affiliations:** ^1^ S.H. Ho Urology Centre, Department of Surgery The Chinese University of Hong Kong Hong Kong China; ^2^ European Association of Urology Section of Endourology (ESEUT) Arnhem The Netherlands; ^3^ Department of Urology Singapore General Hospital Singapore; ^4^ Department of Urology University Hospital Southampton, NHS Trust Southampton UK; ^5^ Department of Urology, Abdul Wahab Sjahranie Hospital Medical Faculty Muliawarman University Samarinda Indonesia; ^6^ Urology Department Menoufia University Shibin el Kom Egypt; ^7^ Department of Urology, Moscow Urology Center Botkin Hospital Moscow Russia; ^8^ Department of Urology Veterans Memorial Medical Center Quezon City Philippines; ^9^ Department of Urology Hospital Alemán de Buenos Aires Buenos Aires Argentina; ^10^ Department of Urology, Ankara University School of Medicine Department of Urology Ankara Turkey; ^11^ Department of Urology Saint Petersburg State University Hospital St. Petersburg Russian Federation; ^12^ Department of Urology Istanbul University Istanbul Faculty of Medicine Istanbul Turkey; ^13^ Xinhua Hospital Shanghai Jiao Tong University School of Medicine Shanghai China; ^14^ Department of Urology American University of Beirut Medical Center Beirut Lebanon; ^15^ Li Ka Shing Institute of Health Sciences The Chinese University of Hong Kong Hong Kong; ^16^ Center for Translational Urological Research, Shenzhen Research Institute The Chinese University of Hong Kong Shenzhen China; ^17^ Department of Urology Medical University of Vienna Vienna Austria; ^18^ Department of Urology Sorbonne University Paris France; ^19^ Department of Urology University of British Columbia Vancouver Canada; ^20^ Urology Unit Azienda Ospedaliero‐Universitaria Delle Marche Ancona Italy; ^21^ Division of Urology Ng Teng Fong General Hospital Singapore Singapore; ^22^ Asian Institute of Nephrology and Urology, AINU India

**Keywords:** FANS, flexible and navigable suction ureteral access sheath, flexible ureteroscopy, long‐term outcomes, nephrolithiasis, ureteric stricture

## Abstract

**Introduction:**

Flexible and navigable suction access sheath (FANS) with flexible ureterorenoscopy (FURS) has demonstrated favourable 30‐day and 3‐month outcomes for renal stones in normal anatomy. We aimed to investigate the long‐term 1‐year safety and efficacy of FANS for renal and ureteric stones in normal or anomalous anatomy.

**Methods:**

This prospective multicentre study included adults undergoing FURS with FANS across 11 centres in 9 countries (April 2023 to August 2024), with follow‐up until August 2025. Anatomical outcomes were assessed by contrast CT (or ultrasound if CT not available) at 1 year; stone‐free rate (SFR) was assessed via noncontrast CT at 30 days. The primary aim was to report if altered anatomy, such as pelvicalyceal or pelviureteric or ureteric stricture, occurs at a later stage.

**Results:**

Among 288 patients, median age was 55 years, and 4.5% had anomalous renal anatomy. About 49% were prestented. Stones were located only in the kidney (62%), ureter (18%) or both (20.1%). Median stone volume was 725 mm^3^; median Hounsfield unit was 1100. Most procedures (84%) used a 7.5Fr scope with 10/12 Fr FANS. Regarding exit strategies, 65% were stented, 14% had an overnight ureteric catheter and 21% were tubeless. Mild bleeding occurred in 17% with no postoperative sepsis, transfusions or persistent hematuria. One patient (0.35%) experienced a Grade 1 ureteric injury. On a 30‐day CT, 82% achieved zero residual fragments (Grade A SFR). On 1‐year CT, five patients (1.7%) were diagnosed with ureteric stricture, 9.7% had persistent residual fragments, and 5.6% developed new ipsilateral stones. Of the five patients with strictures, three had a history of ureteric re‐implantation and only one had been prestented. Serum creatinine showed no significant change from baseline.

**Conclusions:**

This study reports the longest 1‐year follow‐up data for patients undergoing FURS with FANS to date. The study did not identify a high rate of late anatomical complications after successful FANS deployment in the treatment of renal and ureteric stones. Caution is warranted in patients with a history of ureteral re‐implantation due to elevated risk of stricture formation.

## 1 INTRODUCTION

Flexible and navigable suction ureteral access sheath (FANS) has emerged as an innovative tool in flexible ureteroscopy (FURS), designed to enhance stone clearance while mitigating risks associated with elevated intrarenal pressures and temperatures.^1^, ^2^, ^3^ A key characteristic that distinguishes FANS from conventional ureteral access sheaths (UAS) is its flexible, atraumatic distal 10‐ to 12‐cm tip, which can be navigated passively with the deflection of the flexible ureteroscope across the pelviureteric junction (PUJ) into the target calyx for aspiration of stone dust and fragments.

Prior studies have established short‐term (30‐day and 3‐month) safety and efficacy of FANS, with high single‐stage stone‐free rates (SFRs) and low complications in normal renal anatomy.^4^ However, concerns persist regarding potential long‐term anatomical sequelae, particularly PUJ or ureteral complications from sheath manipulation across PUJ and within the pelvicalyceal system (PCS).

While 3‐month data have shown minimal anatomical changes,^4^ no study to date has reported 1‐year outcomes for FANS. The present multicentre study aims to evaluate the long‐term safety, efficacy and anatomical integrity of the upper urinary tract following FURS with FANS at 1‐year follow‐up in a diverse cohort including patients with normal and anomalous renal anatomy.

## 2 PATIENTS AND METHODS

### 2.1 Study design and patient selection

This was a prospective, multicentre study conducted across 11 centres in 9 countries from April 2023 to August 2024, with follow‐up until August 2025. Only adult patients (>18 years) who underwent successful FURS with FANS for renal or proximal ureteral stone were included, irrespective of previous failed attempts, stone size, burden, in normal or anomalous kidneys. Prestenting was not mandatory; patients with elective or emergency prestenting were also included. Ureteral stones, whether impacted or nonimpacted, were eligible provided a FANS was used. Exclusion criteria were as follows: failed FANS deployment, age <18 years, pregnancy, incomplete data records and any combined surgery performed in the same sitting.

Institutional review board approval was obtained, and anonymised data were maintained under an approved registry (CRE‐2021.684).

### 2.2 Data collection and outcomes

Baseline demographics, operative details and follow‐up data up to 1 year were recorded. All patients underwent low‐dose, 2‐mm cut NCCT preoperative and postoperatively at 30 days. Stone features and residual fragments were assessed in the bone window. Stone volume was calculated using the ellipsoid formula (length × width × depth × π × 0.167). Surgeon choice of FURS, FANS and laser source was based on institutional availability. Positive preoperative cultures were treated per local antibiotic sensitivity. Anticoagulant or antiplatelets were stopped 3 days preoperatively and restarted at surgeon's discretion. At 1 year, imaging (CT or ultrasound if CT was unavailable) assessed hydronephrosis, PUJ/PCS anomalies, residual fragments and new stones.

Operative time, ureteroscopy time, laser parameters, sheath details and intra‐operative/postoperative complications were documented.

Stone‐free status on 30‐day CT was graded as follows:

- Grade A: 100% stone‐free, zero residual fragment (ZRF) status
- Grade B: Single RF not more than 2 mm in maximum diameter.
- Grade C: Single RF 2.1–4 mm in maximum diameter.
- Grade D: Single or multiple residual fragments (RF) > 4 mm in maximum diameter.

Operative time was defined as the time from start of cystoscopy to exit strategy (stent, ureteric catheter or nil drainage). Ureteroscopy time was defined from scope insertion to FANS removal. Secondary outcomes of interest included perioperative complications within 24 h, such as bleeding, transfusion, ureteric injury, PCS injury and sepsis defined according to the Third International Consensus (Sepsis‐3). Loin pain score was measured with a standard 10‐point visual analogue score (VAS), with 1 being lowest and 10 being highest at day 1 post‐FURS.

### 2.3 Statistical analyses

All statistical analyses were performed using R Statistical language, version 4.4.2 (R Foundation for Statistical Computing, Vienna, Austria) with *p* < 0.05 indicating statistical significance. Continuous variables were described using median and interquartile range, while categorical variables were described using absolute numbers and percentages. Patient demographics, perioperative parameters and outcomes were compared between sheath sizes using the *χ* ^2^ test or Fisher exact test for categorical parameters and the Mann–Whitney U test for continuous variables.

Finally, multivariable logistic regression analysis was performed to evaluate factors associated with 30‐day 100% stone‐free in this contemporary cohort. Variables which have been suggested in previous literature to impact SFR were entered into a multivariable model to assess their significance as independent predictors. These predictors were described using OR, 95% CI and *p*‐values.

## 3 RESULTS

### 3.1 Baseline and operative characteristics

A total of 288 patients with follow‐up up to 1 year were analysed (Table 1). A total of 22 were excluded due to incomplete data. Median age was 55 years, 57% male and 4.5% (*n* = 13) had altered renal anatomy (seven malrotated kidneys, three duplex ureters and three had prior ureteral reimplantation: two of which received open ureterovesical reimplantation following iatrogenic injury and the remaining had laparoscopic proximal ureteroureterostomy due to unknown cause). All three patients received ureteral surgery more than 10 years prior to FURS with FANS (Table 2).

**TABLE 1:** Baseline characteristics. reported as median [interquartile range] or *N* (%).

	Overall (*n* = 288)
Age	55 [46, 64]
Male sex	163 (57)
DM	56 (19)
HTN	112 (39)
IHD	38 (13)
BMI, kg/m^2^	26.0 [24.0, 28.2]
Blood thinners	
None	251 (87)
Anticoagulant	23 (8.0)
Antiplatelet	14 (4.9)
Normal kidney anatomy	275 (95)
Laterality	
Left	150 (52)
Right	126 (44)
Bilateral	12 (4.2)
First time stone former	164 (57)
Presentation	
Hematuria	19 (6.6)
Pain	197 (68)
Fever	30 (10)
Incidental	39 (14)
Hematuria and pain	3 (1.0)
Positive urine culture	53 (18)
Preoperative antibiotics	288 (100)
Prestented	142 (49)
Failure in primary FANS deployment	26 (9.0)
Routine practice	38 (13)
Emergency	78 (27)
Stone location	
Renal upper or middle pole	73 (25)
Renal lower pole	82 (28)
Renal pelvis	23 (8.0)
Ureter only	52 (18)
Multiple stones, all renal	34 (12)
Multiple stones, renal and ureteric	24 (8.3)
Hounsfield units (all)	1100 [843, 1309]
Stone volume, mm^3^ (all)	725 [339, 1343]
Hounsfield units (kidney)	1095 [814, 1285]
Stone volume, mm^3^ (kidney)	820 [378, 1363]
Hounsfield units (ureter)	1110 [762, 1365]
Stone volume, mm^3^ (ureter)	415 [258, 993]
Scope size	
Mini (7.5Fr or smaller)	242 (84)
Standard (larger than 7.5Fr)	46 (16)
Laser	
Low‐power Holmium (<40w)	54 (19)
High‐power Holmium (Lumenis or Quanta)	100 (35)
Thulium fibre (CyberHo 75 or IPG 40 W)	112 (39)
Pulsed Thulium‐YAG (Dornier Thulio)	21 (7.3)
No laser used, only suction via FANS	1 (0.3)

**TABLE 2:** Intraoperative and postoperative complications. reported as median [interquartile range] or *N* (%).

	Overall (*n* = 288)
Mild bleeding without need for blood transfusion (CD1)	49 (17)
Case abandoned	0
Ureteric injury	1 (0.35)
Pelvicalyceal system injury due to sheath placement	0
Intraoperative/postoperative transfusion (CD2)	0
Postoperative fever > 38 C managed with antibiotics (CD2)	2 (0.69)
Postoperative sepsis not needing ICU (CD2)	0
Postoperative sepsis requiring ICU stay (CD4)	0
Persistent postoperative hematuria	0
Persistent loin pain	0
Arteriovenous malformation requiring embolisation	3 (1.0)
Pain score (VAS 1–10, 1 being lowest)	1 [0, 3]
Hospital stay, days	1 [0, 2]
Reintervention for residual fragment	1 (0.35)
Residual fragment grade on 30‐day CT	
A—Zero RF	236 (82)
B—Single RF < 2 mm	30 (10)
C—Single RF 2–4 mm	9 (3.1)
D—Single RF > 4 or any size multiple	13 (4.5)
1‐year outcomes on CT	
PCS anomaly	0
PUJ anomaly	0
Ureteric stricture	5 (1.7)
Change in serum creatinine	0.0 [−0.1, 0.0]
Residual fragment present at 1‐year CT (Grade C + D)	28 (9.7)
Diameter, mm	3 [2, 6]
New ipsilateral stone	16 (5.6)
Mortality	0

Abbreviations: CD, Clavien Dindo classification; CT, computer tomography; PCS, pelvicalyceal system; PUJ, pelviureteric junction; RF, residual fragment.

Median stone volume was 725 mm^3^. The most common sheath size was 10/12 Fr. About 84% used a flexible ureteroscope size 7.5Fr or smaller. Thulium fibre laser was used in 39% of cases. Exit strategies included stenting (65%), overnight catheter (14%) and tubeless (21%).

### 3.2 Perioperative outcomes

Intraoperative mild bleeding due to sheath movement occurred in 17%. One ureteral injury (0.35%) (Grade 1 Thomas‐Traxer) was reported. No cases of sepsis or transfusion were reported. The 30‐day NCCT revealed 82% Grade A SFR (ZRF) and 92% Grade A + B SFR (Table S2).

### 3.3 The 1‐year outcomes

All patients received a CT scan at 1 year. Five patients (1.7%) developed ureteral strictures, all diagnosed on follow up CT (Table 3). Only one stricture patient was prestented. Three had prior ureteral reimplantation. Residual fragments (Grades C and D) were present in 9.7% of patients, and 5.6% developed new ipsilateral stones. No new PUJ or PCS anomalies were reported. Serum creatinine levels remained stable.

**TABLE 3:** Characteristics of five patients with ureteric stricture. reported as median [interquartile range] or *N* (%).

	Overall (*n* = 5)
Age, mean ± SD	54 ± 8
Male sex	5 (100)
Blood thinners	0
Abnormal kidney anatomy (all three were re‐implanted ureters)	3 (60)
Prestented	1 (20)
Stone location	
Renal upper or middle pole	1 (20)
Renal lower pole	3 (60)
Ureter only	1 (20)
Hounsfield units (kidney)	1187 [1187, 1285]
Stone volume, mm^3^ (kidney)	4111 [4071, 4111]
Hounsfield units (ureter)	1452
Stone volume, mm^3^ (ureter)	252
Scope size	
Mini (7.5Fr or smaller)	4 (80)
Standard (larger than 7.5Fr)	1 (20)
Laser	
Low‐power Holmium (<40w)	1 (20)
High‐power Holmium (Lumenis or Quanta)	1 (20)
Thulium fibre (CyberHo 75 or IPG 40 W)	3 (60)
Laser settings	
Dusting, J	0.2 [0.2, 0.5]
Dusting, Hz	30 [20, 30]
Popcorning, J	0.5 [0.5, 0.6]
Popcorning, Hz	30 [25, 30]
Ureteroscopy time	20 [20, 28]
Total operation time	28 [28, 32]
Sheath able to access kidney	5 (100)
Sheath able to access lower pole	1 (20)
Residual fragment present at 30 days	3 (60)
Change in serum creatinine, mean ± SD	0.13 ± 0.14
Diagnosis of stricture on CT	5 (100)
Stricture location	
Proximal ureter	3 (60)
Mid‐ureter	2 (40)
Treatment	
Balloon dilation	3 (60)
Laser endoureterotomy	1 (20)
Stenting	1 (20)

### 3.4 Predictors of 30‐day stone‐free status

Multivariable analysis (Table S1) identified older age, lower stone volume and use of Magneto holmium pulse modulated laser as independent predictors of 30‐day ZRF. Lower pole and multiple renal stones were negative predictors.

## 4 DISCUSSION

The recent development of suction‐assisted technology—specifically, Flexible and Navigable Suction Ureteral Access Sheaths (FANS)—represents a major advancement in the field of FURS.^5^ Initial randomised controlled trials have consistently shown that FANS offers several short‐term benefits compared to conventional UAS at 3 months, such as higher SFRs, fewer postoperative infections, improved patient quality of life and reduced use of stone baskets.^2^, ^6^ Notably, the first international multicentre RCT with similar numbers of prestented patients in both FANS and conventional UAS groups (11.9% vs. 13.8%, *p* = 0.616) reported a greater incidence of ureteral wall injuries with conventional UAS than with FANS (Grade I: 35% vs. 11.9%). Zhu et al. attributed this to FANS's smoother placement, which encounters less resistance, and its flexible, atraumatic tip.^2^

The principal finding of this study is the sustained anatomical safety of FANS at 1 year, as evidenced by a remarkably low overall ureteral stricture rate of 1.7% (5/288 patients). This is comparable to the historical rates of 0.1%–4.9% following ureteroscopy and can rise up to 34% in patients with impacted ureteral stones.^7^ Ureteral strictures complications are well‐documented even in the international multicentre FLEXOR registry, where FURS was used with a conventional UAS.^8^

The long‐term anatomical consequences of navigating a FANS across the PUJ and within the PCS have remained a point of clinical inquiry and concern. This study represents the longest follow‐up to date for FURS with FANS, demonstrating sustained safety and efficacy at 1 year. Of note, the five strictures detected in our cohort were located at the proximal ureter (60%) and mid‐ureter (40%) in our cohort (Table 3). This mitigates earlier theoretical concerns that the unique design of FANS—which allows for active navigation and suction within the PCS—might impart undue mechanical stress or ischaemic insult at the PUJ, a vulnerable anatomical watershed area. Our data suggest that when FANS is used with proper technique,^9^ FANS does not confer an additional risk for acquired PUJ obstruction.

Notably, three of the five patients who developed a stricture in our cohort had a history of ureteral reimplantation, suggesting that altered ureteral anatomy or prior surgical trauma may increase vulnerability. This subgroup represented a small fraction of our total cohort (only 13 patients had anomalous anatomy), yet it accounted for most of the significant long‐term complications. This finding carries substantial clinical implications. Patients with reimplanted ureters often have altered vascular supply, compromised periureteral tissue and potentially a history of prior stricture disease, rendering them more susceptible to ischemic injury even with a minor mechanical or thermal insult.^10^ Furthermore, only one of these five who developed a stricture was prestented, a practice known to facilitate sheath insertion and potentially reduce traumatic injury.^11^ Similar to guidelines, while our overall data do not mandate universal prestenting, this observation in high‐risk anatomy suggests that a more cautious approach, including routine prestenting and consideration of using the smallest effective sheath size, may be prudent in patients with a history of ureteral surgery.

The stone‐free outcomes in this study further cement the efficacy profile of FANS. The 30‐day ZRF rate of 82% and Grade A + B SFR of 92% are excellent, especially considering the cohort included complex cases such as lower pole stones (28%) and multiple stones (20%). These results are consistent with and even surpass those reported in the initial global FANS study and other comparative trials.^1^, ^2^, ^12^ The high immediate SFR is a direct benefit of the continuous suction, which maintains a clear visual field, reduces stone retropulsion and actively evacuates dust and small fragments. This efficiency translates into durable success, with a reintervention rate of only 0.35% at 1 year for residual fragments, in contrast to the contemporary reintervention rates of 51.5% within 3 months in those 21.7% who have residual fragments after FURS without the use of suction in the real‐world FLEXOR registry.^13^ Notably, the 5.6% rate of new ipsilateral stone formation is in line with the known recurrence rates for urolithiasis and is unlikely related to the sheath technology itself but rather to patient‐specific metabolic factors.

The operative data underscore the versatility and user‐acceptance of FANS in real‐world practice. The majority of cases (84%) utilised a mini ureteroscope (= < 7.5Fr) with a 10/12 Fr sheath, reflecting a trend towards minimising ureteral trauma while still achieving effective suction. The high rates of tubeless procedures (21%) and overnight catheter only strategies (14%) reflect growing surgeon confidence in the atraumatic nature of the procedure and the reduced fear of postoperative obstruction due to the ability to clear all fragments from all locations in the PCS reachable by the FANS.^14^ The low incidence of significant intraoperative complications—only one ureteral injury (0.35%) and no septic events—reinforces the strong safety profile established in shorter‐term studies. The predominance of thulium fibre laser (TFL) use (39%) reflects the contemporary shift in laser lithotripsy and suggests good compatibility between TFL's high ablation efficiency and FANS's suction capabilities for maintaining a low‐temperature, low‐pressure environment.

This study has several limitations. First, the lack of a control group treated with conventional UAS limits our ability to make direct comparative claims about long‐term stricture rates. However, given the robust existing level 1 evidence demonstrating the short‐term superiority of FANS, a randomised long‐term trial comparing suction versus nonsuction UAS may be ethically and practically challenging.^15^ Second, while we mandated imaging at 1 year, such rigorous radiographic follow‐up may not be feasible or justified due to cost and radiation exposure in real‐world practice. Our protocol, however, was necessary to answer the specific research question regarding anatomical sequelae. Whether this now serves as a guide to follow up all FURS where a FANS was used is to be researched, keeping cost and value benefit in perspective. Third, this study population differs from the previously published global FANS study^1^ where only patients with normal anatomy underwent FANS in expert hands. Small sample studies have reported use of FANS in malrotated and anomalous anatomies,^16^ but to our knowledge, this may be the first to report FANS usage in reconstructed/reimplanted ureters, which were the majority of the new onset strictures. Finally, while a multitude of factors can influence immediate SFRs, proper follow‐up is needed to establish long‐term sequelae. Selection bias can be a confounding factor for interpretation as those with failed FANS deployment were excluded. One year is sufficiently long for any ureteral stenosis or stricture to manifest, and longer follow‐up may strain resources; thus, it may be reserved for high‐risk stricture populations where a high index of suspicion is warranted.

The overall low stricture rate in this study instills confidence that FANS, used with appropriate expertise, represents a significant advancement in FURS. Ongoing design modifications and miniaturisation of the FANS sheath may further enhance its safety profile.^3^

Looking forward, several avenues for further research emerge. As the technology evolves, investigating the “safety threshold” for FANS insertion force—potentially using sensor‐integrated sheaths—could provide an objective metric to prevent traumatic injuries, especially in training settings.^17^

Our inference and message from this 1‐year multicentre prospective study provide compelling evidence that FURS with FANS is not only effective in achieving high immediate SFRs but also safe in the long term, with a very low incidence of ureteral stricture and no evidence of iatrogenic PUJ or PCS damage. However, outcomes—both good and bad—are ultimately in the hands of the end‐user, namely, the surgeon, and we advocate caution when using FANS in altered renal and ureteral anatomy. The technology has proven adaptable across various scopes, laser platforms and clinical settings. The caveat lies in its application where the risk–benefit ratio is carefully considered, and caution is exercised.

## 5 CONCLUSION

This study presents the analysis of 1‐year follow‐up data for patients undergoing FURS with FANS, representing the longest follow‐up period reported to date. The study did not identify a high rate of late anatomical complications after successful FANS deployment in the treatment of renal and ureteric stones. However, it is important to exercise caution in patients with a history of ureteral reimplantation, as these individuals may be at increased risk for stricture formation.

## Supporting information


**Table S1.** Multivariable analysis evaluating factors that affect the achievement of zero residual fragments (Grade A stone‐free status) on 30‐day CT.
**Table S2.** Operative characteristics.

## Data Availability

The data that support the findings of this study are available from the corresponding author upon reasonable request.

## References

[1] Gauhar V , Traxer O , Castellani D , Sietz C , Chew BH , Fong KY , et al. Could use of a flexible and navigable suction ureteral access sheath be a potential game‐changer in retrograde intrarenal surgery? Outcomes at 30 days from a large, prospective, multicenter, real‐world study by the European Association of Urology urolithiasis section. Eur Urol Focus. 2024;10(6):975–982. 10.1016/j.euf.2024.05.010 38789313

[2] Zhu W , Liu S , Cao J , Wang H , Liang H , Jiang K , et al. Tip bendable suction ureteral access sheath versus traditional sheath in retrograde intrarenal stone surgery: an international multicentre, randomized, parallel group, superiority study. EClinicalMedicine. 2024;74:102724. 10.1016/j.eclinm.2024.102724 39070176 PMC11277316

[3] Yuen SKK , Traxer O , Wroclawski ML , Gadzhiev N , Chai CA , Lim EJ , et al. Scoping review of experimental and clinical evidence and its influence on development of the suction ureteral access sheath. Diagnostics (Basel). 2024;14(10):1034. 10.3390/diagnostics14101034 38786332 PMC11120421

[4] Fong KY , Yuen SKK , Somani BK , Malkhasyan V , Tanidir Y , Persaud S , et al. Assessment of outcomes and anatomical changes in the upper urinary tract following flexible ureteroscopy with a flexible and navigable suction ureteral access sheath: 3‐month results from a multicenter study. Urology. 2025;199:35–41. 10.1016/j.urology.2025.01.029 39826806

[5] Gauhar V , Castellani D , Tsaturyan A , Taguchi K , Herrmann T , Somani B , et al. Does the existing evidence on flexible and navigable suction ureteral access sheath indicate a potential paradigm shift in the management of kidney and ureteral stones with flexible ureteroscopy? An overview from EAU endourology. Curr Opin Urol. 2025;36(1):35–41. 10.1097/MOU.0000000000001333 40923118

[6] Cacciatore L , Minore A , Bonanno L , Contessa P , Esperto F , Iannello AR , et al. Is flexible navigable suction ureteral access sheath (FANS) safer and more efficient than conventional sheaths? Italian multicentric experience. World J Urol. 2025;43(1):153. 10.1007/s00345-025-05520-9 40050461

[7] Moretto S , Saita A , Scoffone CM , Talso M , Somani BK , Traxer O , et al. Ureteral stricture rate after endoscopic treatments for urolithiasis and related risk factors: systematic review and meta‐analysis. World J Urol. 2024;42(1):234. 10.1007/s00345-024-04933-2 38613692

[8] Giulioni C , Fuligni D , Brocca C , Ragoori D , Chew BH , Emiliani E , et al. Evaluating the safety of retrograde intrarenal surgery (RIRS): intra‐ and early postoperative complications in patients enrolled in the global multicentre flexible Ureteroscopy outcome registry (FLEXOR). Int Braz J Urol. 2024;50(4):459–469. 10.1590/S1677-5538.IBJU.2024.0055 38743064 PMC11262716

[9] Gauhar V , Ong CSH , Traxer O , Chew BH , Gadzhiev N , Teoh JYC , et al. Step‐by‐step guide to flexible and navigable suction ureteric access sheath (FANS). Urol Video J. 2023;20:100250. 10.1016/j.urolvj.2023.100250

[10] Zhang J , Xue W , Tian P , Zheng J , Ding C , Li Y , et al. Effect of ureteral stricture in transplant kidney and choice of treatment on long‐term graft survival. Int Urol Nephrol. 2023;55(9):2193–2203. 10.1007/s11255-023-03669-z 37308613 PMC10406706

[11] Yuen SKK , Castellani D , Tokas T , Pirola GM , Giulioni C , Kwok JL , et al. The influence of pre‐stenting and drugs on the outcomes of ureteroscopy for kidney and ureteral stone disease: a systematic review and meta‐analysis by the EAU section of endourology. World J Urol. 2025;43(1):489. 10.1007/s00345-025-05848-2 40797029 PMC12343641

[12] Zeng G , Jiang K , Liu S , et al. Flexible ureteroscopy with a flexible and navigable suction ureteral access sheath versus mini‐percutaneous nephrolithotomy for treatment of 2‐3 cm renal stones: an international, multicenter, randomized, noninferiority trial. Eur Urol. 2025. 10.1016/j.eururo.2025.06.001 40533283

[13] Gauhar V , Chew BH , Traxer O , Tailly T , Emiliani E , Inoue T , et al. Indications, preferences, global practice patterns and outcomes in retrograde intrarenal surgery (RIRS) for renal stones in adults: results from a multicenter database of 6669 patients of the global FLEXible ureteroscopy outcomes registry (FLEXOR). World J Urol. 2023;41(2):567–574. 10.1007/s00345-022-04257-z 36536170

[14] Castellani D , Somani BK , Fong KY , Yuen SKK , Heng CT , Elshazly M , et al. Can flexible ureteroscopy using flexible and navigable suction ureteral access sheath (FANS‐UAS) minimize postoperative double J stent placement? Results from a propensity score‐matched analysis of 540 patients of the European Association of Urology Section of endourology and global FANS collaborative study group. Investig Clin Urol. 2025;66(3):236–244. 10.4111/icu.20250071 PMC12058539 40312903

[15] Mazzon G , Dewan S , Rasheed M , Kulkarni S , Brusa D , Fontana F , et al. Flexible ureteroscopy for renal stones comparing non suction conventional UAS vs flexible and navigable suction ureteral access sheaths in a multicenter real‐world experience. Is it finally time to bury the no suction ureteral access sheath? An EAU endourology analysis. World J Urol. 2025;43(1):390. 10.1007/s00345-025-05748-5 40560463

[16] Edmonds VS , Ballantyne CC , Cabo JJ , Ostergar AS , Humphreys MR , Stern KL . First prospective, in vivo experience using a novel steerable suction ureteral access sheath in North America. Urology. 2025;205:38–45. 10.1016/j.urology.2025.07.064 40754021

[17] Tefik T , Ergul RB , Osther P , et al. The relationship between the force applied and perceived by the surgeon during ureteral access sheath placement: ex‐vivo experimental model. World J Urol. 2024;42(1):329. 10.1007/s00345-024-04982-7 38753120 PMC11098873

