# Supplementary material for: Assessment of anatomical outcomes in the upper urinary tract following flexible ureteroscopy with flexible and navigable suction ureteral access sheath: 1‐year results from a multicentre study
Source: BJUI Compass. 2026 May 4;7(5):e70213. doi: 10.1002/bco2.70213 (PMC13138902; doi:10.1002/bco2.70213)
Supplement: Supplementary file 1 — Table S1. Multivariable analysis evaluating factors that affect the achievement of zero residual fragments (Grade A stone‐free status) on 30‐day CT. Table S2. Operative characteristics. [file BCO2-7-e70213-s001.docx]

# **Supplementary Table 1**. Multivariable analysis evaluating factors that affect the achievement of zero residual fragments (Grade A stone-free status) on 30-day CT

| Variable | OR (95%CI | p value |
| --- | --- | --- |
| Age | 0.95 (0.92 – 0.97) | 0.001 |
| Male sex (vs female) | 1.59 (0.77 – 3.29) | 0.2 |
| Stone location (vs renal upper/middle pole) |  |  |
| Renal lower pole | 0.20 (0.06 – 0.63) | 0.008 |
| Renal pelvis | 0.69 (0.12 – 4.50) | 0.7 |
| Ureter only | 0.88 (0.19 – 4.08) | 0.9 |
| Multiple stones, all renal | 0.22 (0.05 – 0.93) | 0.043 |
| Multiple stones, renal and ureteric | 0.34 (0.07 – 1.58) | 0.17 |
| Stone volume | 0.56 (0.40 – 0.79) | 0.001 |
| Prestented | 1.11 (0.52 – 2.41) | 0.8 |
| Standard size scope (vs mini) | 1.32 (0.37 – 5.03) | 0.7 |
| Laser (vs low-power Holmium) |  |  |
| High-power Holmium | 1.10 (0.27 – 4.41) | 0.9 |
| Thulium fiber | 1.24 (0.32 – 4.70) | 0.8 |
| Magneto | 12.45 (1.43 – 284) | 0.043 |
| Pulsed Thulium-YAG | 0.88 (0.15 – 5.79) | 0.9 |

**Supplementary Table 2. Operative characteristics.**Reported as median [interquartile range] or N (%).

|  | **Overall**  (n=288) |
| --- | --- |
| Anesthesia |  |
| General | 188 (65) |
| Spinal | 99 (34) |
| Local with sedation | 1 (0.35) |
| Scope size |  |
| Mini (7.5Fr or smaller) | 242 (84) |
| Standard (larger than 7.5Fr) | 46 (16) |
| Laser |  |
| Low-power Holmium (<40w) | 54 (19) |
| High-power Holmium (Lumenis or Quanta) | 71 (25) |
| Thulium fiber (CyberHo 75 or IPG 40W) | 112 (39) |
| Magneto (Quanta 100W) | 29 (10) |
| Pulsed Thulium-YAG (Dornier Thulio) | 21 (7.3) |
| No laser used, only suction via sheath sufficient to clear stone | 1 (0.35) |
| Laser settings |  |
| Dusting, J | 0.5 [0.4, 0.8] |
| Dusting, Hz | 20 [14, 30] |
| Popcorning, J | 1.0 [1.0, 1.5] |
| Popcorning, Hz | 10 [10, 20] |
| Stone fragmentation modality |  |
| Dusting | 126 (44) |
| Fragmentation | 51 (18) |
| Combination | 109 (38) |
| Basket for stone repositioning |  |
| Suction type |  |
| Wall-mounted | 73 (25) |
| Floor-mounted | 162 (56) |
| Automated pressure suction | 53 (18) |
| Irrigation device type |  |
| Gravity | 60 (21) |
| Gravity + pressure cuff | 213 (74) |
| Endoflow | 14 (4.9) |
| Automated | 1 (0.35) |
| Ureteroscopy time | 32 [26, 46] |
| Total operation time | 42 [32, 62] |
| Scope damage |  |
| None | 278 (97) |
| Damaged but can continue use | 7 (2.4) |
| Damaged, requires replacement | 3 (1.0) |
| Sheath damage |  |
| None | 281 (98) |
| Damaged but can continue use | 7 (2.4) |
| Damaged, requires replacement | 0 |
| Sheath able to access all of kidney | 262 (95) |
| Sheath able to access lower pole | 214 (88) |
| Exit strategy |  |
| Stent, no thread | 143 (50) |
| Stent, with thread | 43 (15) |
| Overnight ureteric catheter | 40 (14) |
| Totally tubeless | 59 (21) |
